# T-2 Toxin-Induced Hepatotoxicity in HepG2 Cells Involves the Inflammatory and Nrf2/HO-1 Pathways

**DOI:** 10.3390/toxins17080397

**Published:** 2025-08-08

**Authors:** Mercedes Taroncher, Felipe Franco-Campos, Yelko Rodríguez-Carrasco, María-José Ruiz

**Affiliations:** 1 Research Group in Alternative Methods for Determining Toxics Effects and Risk Assessment of Contaminants and Mixtures (RiskTox), University of Valencia, 46100 Valencia, Spain; felipe.franco@uv.es (F.F.-C.); yelko.rodriguez@uv.es (Y.R.-C.); m.jose.ruiz@uv.es (M.-J.R.); 2 Laboratory of Food Chemistry and Toxicology, Faculty of Pharmacy and Food Sciences, University of Valencia, Av. Vicent Andrés Estellés s/n, 46100 Valencia, Spain

**Keywords:** T-2 toxin, oxidation, inflammation, immunofluorescence, qPCR, Western blot

## Abstract

The T-2 toxin is one of the most toxic mycotoxins, to which the population is exposed through the diet. T-2 toxins are especially found in cereals and cereal-based products. To deepen our understanding of the mechanisms of T-2 toxin action, the morphological changes, oxidative stress, and inflammatory response of this mycotoxin have been evaluated in HepG2 cells. The mRNA and protein expression levels of inflammatory cytokines such as IL-1β, IL-6, and TNF-α and proteins such as Nrf2 and HO-1 were analyzed after T-2 exposure (7.5, 15, and 30 nM) by qPCR and Western blot assays. Firstly, changes in the morphology of HepG2 cells after T-2 exposure from circular to elongated shape were observed in a concentration-dependent manner by indirect immunofluorescence. These alterations may reflect early signs of cell stress. The results revealed an upregulation of the mRNA of IL-1β, IL-6, and TNF-α after T-2 exposure, with the highest increase in TNF-α after 30 nM T-2, suggesting a proinflammatory effect. Regarding the oxidative response, HO-1 at the lowest T-2 concentration was upregulated. However, the Nrf2 at all T-2 concentrations tested was downregulated. These findings were corroborated by Western blot analysis. These results confirm that T-2 hepatotoxicity produces an increase in key inflammatory cytokines, modulates the Nrf2/HO-1 pathway, and produces morphological changes in HepG2 cells. The next step would be to test whether a co-exposure of natural antioxidants with T-2 exerts a cytoprotective effect.

## 1. Introduction

Mycotoxins are toxic metabolites produced by certain species of fungi of the genera *Fusarium*, *Aspergillus*, *Penicillium*, *Alternaria*, and *Claviceps*. To date, over 900 mycotoxins have been identified, many of which involve a significant threat to both animal and human health [1]. Despite regulatory efforts to reduce fungal contamination during pre- and post-harvest stages, effective control remains a considerable challenge. These compounds are difficult to eliminate due to their chemical stability and resistance to conventional food processing techniques, making them a persistent concern for food and feed safety [2]. As exposure to mycotoxins is inevitable, it is essential to investigate their potential health effects in humans. The T-2 toxin (T-2) is a *Fusarium* mycotoxin belonging to the trichothecenes group produced by *F*. *poae*, *F*. *acuminatum*, *F*. *sporotichioides*, *and F*. *equiseti* species. This mycotoxin is the most common and cytotoxic of the group A trichothecenes. The main T-2 exposure in humans is through dietary ingestion [3]. The occurrence of T-2 and its main metabolite, the HT-2 toxin, is frequently reported in wheat, maize, barley, oats, and their processed foods. The HT-2 toxin has similar toxicological properties than T-2 [4]. The mechanisms of toxic action of these mycotoxins have been studied over the years. Nonetheless, the wide variability in the presence of T-2 in food products has reignited discussion about its toxicological relevance, and the need to re-evaluate its mechanisms of action to better elucidate its toxicokinetics, adverse health effects, and associated risks. Among its mechanisms of action, T-2 has been shown to induce organ damage to the kidneys, skin, liver, hematopoietic, brain, gastrointestinal, and reproductive systems [5,6]. However, its impact on the immune system, particularly the induction of inflammatory cytokine production in hepatic cells, is not yet fully elucidated and warrants further investigation. Cytokines are essential for the growth and development of mammals. Many developmental, physiological, and immunological issues can arise from the abnormal expression of the genes encoding cytokines. It has been reported that T-2 can either activate or depress the immune system, depending on the dose, frequency of exposure, timing, and functional immunological assay used [5]. Proinflammatory cytokines, such as TNF-α, IL-1β, IL-6, and IL-8, play an important role in developing immune responses and thereby acute inflammation. In particular, T-2 exposure has been shown to induce a dose-dependent increase in IL-8 production in Caco-2 cells, suggesting a potential role in intestinal inflammation [6]. According to Wu and colleagues, oxidative stress is a critical mediator of immunotoxicity. Inflammation in different tissues caused by T-2 is associated with oxidative stress, ROS activation, lipid peroxidation, and increased levels of inflammatory cytokines [5]. One crucial defense mechanism against oxidative damage in cells is redox balance. One significant cytoprotective factor is Nrf2. It regulates the antioxidant defense system through its ability to bind to antioxidant response elements (AREs). Furthermore, Nrf2 activates genes encoding cytoprotective enzymes, such as catalase (CAT), superoxide dismutase (SOD), glutathione peroxidase (GPX), and NAD(P)H quinone dehydrogenase 1 (NQO1) as well as, notably, heme oxygenase-1 (OH-1). HO-1 is recognized for its dual functionality, contributing to redox homeostasis and also exerting regulatory effects on inflammation-related signaling cascades [7,8,9]. The Nrf2 pathway plays a crucial part in reducing oxidative damage by regulating the cellular response to harmful compounds [3]. Activation of the Nrf2/HO-1 pathway is therefore considered a significant mechanism by which cells attempt to restore redox balance and limit inflammation [10]. The Nrf2/HO-1 signaling pathway has a protective role against oxidative stress induced by T-2 and serves as central focus in the investigation of the oxidative stress defense mechanism in this study [11]. Moreover, oxidative stress may be related to the production of proinflammatory cytokines. Although T-2-induced cytokine production has been reported in various cell types, including macrophages, neurons, and human placental epithelial cells [2,12,13], this inflammatory response has not yet been explored in HepG2 cells.

A deeper understanding of the molecular pathway through which T-2 induced both inflammation and oxidative stress is therefore of particular interest. In this line, developing strategies to prevent or mitigate the inflammatory response may represent a promising approach to counteracting T-2-induced toxicity.

The liver is involved in numerous metabolic processes, including the biotransformation of a wide variety of toxic substances. It is also the primary organ for the metabolism and detoxification of the T-2 toxin [4,14,15]. Accordingly, liver-derived cell lines, such as HepG2, are widely recognized as relevant in vitro models for toxicological studies [16]. Other studies have described that T-2 exposure increases oxidative stress in various cell lines, potentially leading to cellular damage and inflammatory responses [17,18,19,20,21]. However, to the best of the authors’ knowledge, no studies have simultaneously evaluated the effects of T-2 on both oxidative stress and proinflammation cytokine production in HepG2 cells.

Therefore, this work aims to investigate T-2-induced hepatotoxicity through inflammatory signaling and oxidative stress by measuring the inflammatory markers and protein gene expression by qPCR and confirming the results through Western blot in HepG2 cells. The findings from this study are expected to provide novel insights that may contribute to the improved monitoring of food contamination and the prevention of T-2-induced liver inflammation.

## 2. Results

### 2.1. Analysis of HepG2 Morphology

**Figure 1:**
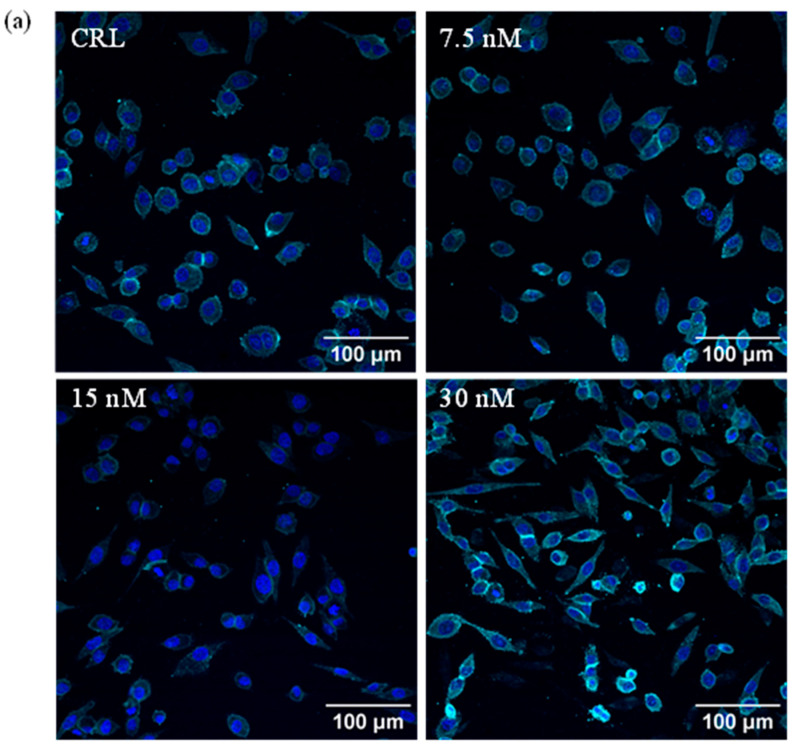

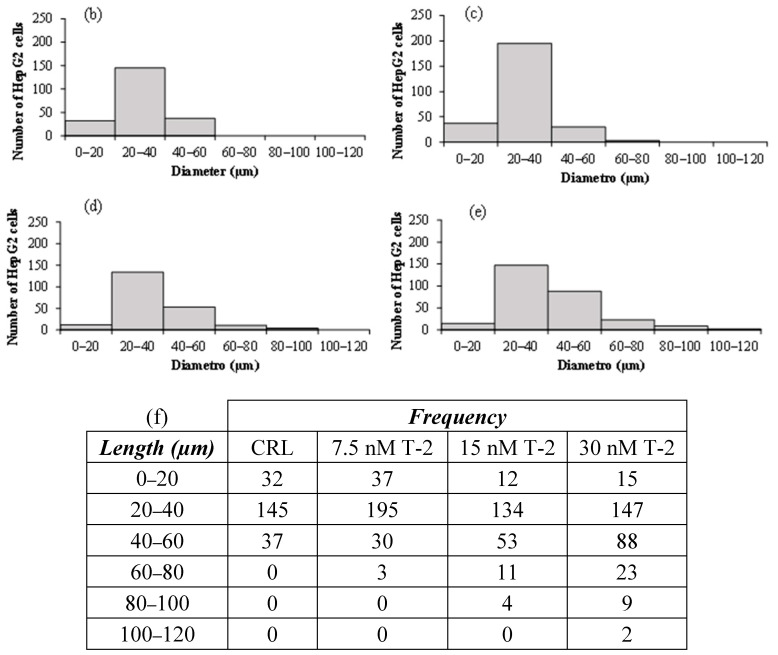
Effect of T-2 on the morphology of HepG2 cells. (**a**) Exposure of HepG2 cells to 7.5, 15, and 30 nM of T-2 for 24 h was analyzed by indirect immunofluorescence. The nucleus was stained with DAPI (blue) and β-actin (cyan). Cells were photographed at 20× with 1.5 digital zoom. Scale bars are 100 μm. (**b**–**e**) The HepG2 cells’ diameter (μm) at CRL, 7.5, 15, and 30 nM of T-2 exposure, respectively. (**f**) Frequency distribution of HepG2 cell sizes after CRL, 7.5, 15, and 30 nM of T-2 exposure. The mean ± SEM of three independent experiments (*n* = 3) is used to express the data. CRL: control.

**Figure 2:**
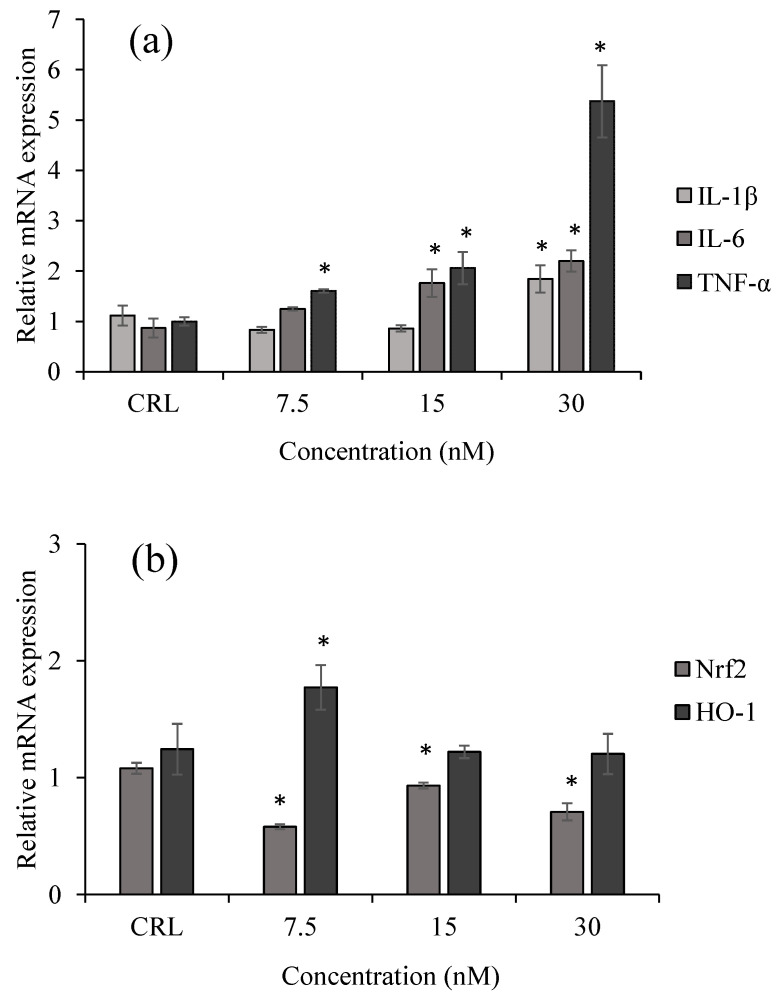
Effect of 7.5, 15, and 30 nM of T-2 on IL-1β, IL-6, and TNF-α (**a**) and Nrf2 and HO-1 (**b**) expression in HepG2 cells after 24 h. Real-time qPCR was used to measure the relative mRNA expression levels. The corresponding 18S rRNA value was used to standardize the average of the target gene values and is expressed as a fold change compared to the solvent control. The mean ± SEM of three independent experiments (*n* = 3) is used to express the data. (*) *p* ≤ 0.05 indicates significant differences compared to the control. CRL: control.

Morphological changes in HepG2 cells exposed to 7.5, 15, and 30 nM of T-2 for 24 h were evaluated by indirect immunofluorescence, as shown in Figure 1. Control cells were exposed to the solvent control (≤1% MeOH) over 24 h. As shown in Figure 1, T-2 induced a concentration-dependent morphological shift, with cells transitioning from a rounded shape to a larger and more elongated form. Figure 1b–e represent the distribution of cell counts based on cell diameter for each condition (CRL, 7.5, 15, and 30 nM T-2). The frequency distribution of cell size is detailed in Figure 1f.

### 2.2. Inflammatory and Oxidative Response of HepG2 Exposed to T-2 by qPCR

To assess the correlation between the cytotoxic effect of T-2 and the induction of inflammatory or oxidative responses, its impact on the expression of proinflammatory genes (IL-1β, IL-6, and TNF-α) and oxidative-stress-related genes (Nrf2 and HO-1) was evaluated in HepG2 cells. Evaluation of the relative mRNA expression levels of inflammatory and oxidative genes in HepG2 cells exposed to 7.5, 15, and 30 nM of T-2 showed that after 24 h of exposure, a proinflammatory response was produced (Figure 2). The IL-1β mRNA was only upregulated at the highest concentration tested (30 nM of T-2) up to 1.84 ± 27-fold compared to the control. The IL-6 was upregulated at 15 and 30 nM of T-2 up to 2.20 ± 21-fold, compared to the control. The TNF-α mRNA increased at all concentrations reaching a 5.37 ± 0.72-fold increase, compared to the control (Figure 2a). Similarly, HO-1 mRNA levels were upregulated up to 0.43 folds compared to the control at the lowest concentration of T-2. However, the Nrf2 mRNA decreased up to 1.77 ± 0.19-folds by T-2 at all concentrations tested, respective to the control (Figure 2b).

### 2.3. Effects of T-2 on Inflammatory and Oxidative Proteins by Western Blot

**Figure 3:**
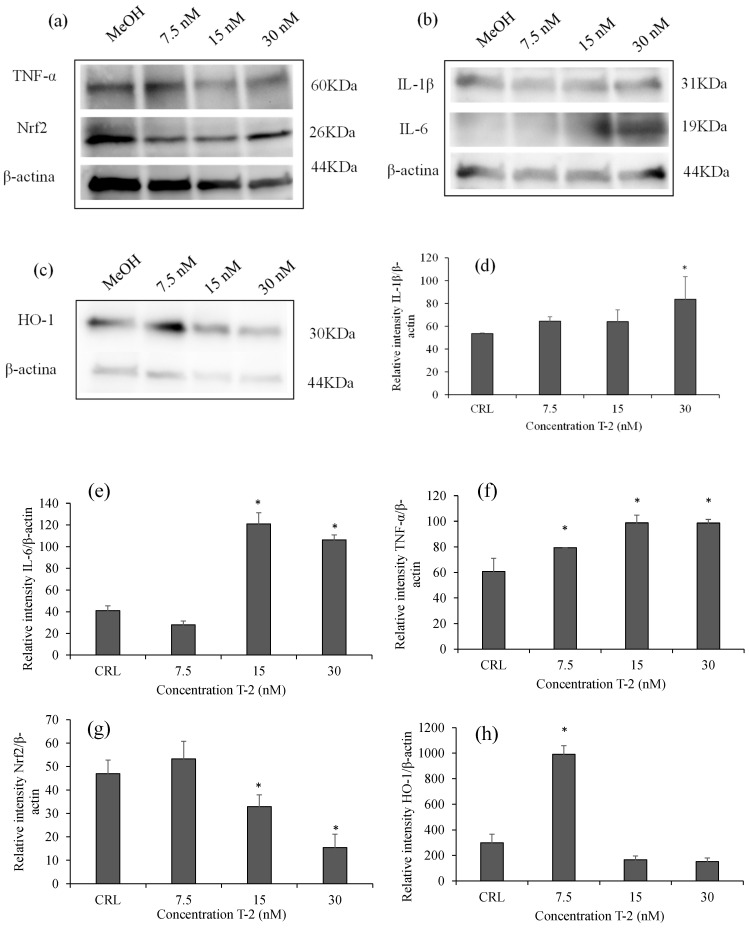
Western blot showing the expression levels of IL-1β, IL-6, TNF-α, Nrf2, and HO-1 proteins and their densitometric analysis in the lysates of HepG2 cells after 7.5, 15, and 30 nM of T-2 over 24 h. (**a**–**c**) Western blot band detection against IL-1β, IL-6, TNF-α, Nrf2, and HO-1 factors and the β-actin reference protein (44 kDa). (**d**–**h**) Densitometric analysis for IL-1β, IL-6, TNF-α, Nrf2, and HO-1 factors/β-actin vs. the control. To express the relative intensity of each factor/β-actin, the average of the target protein value was normalized to the matching β-actin value. The mean ± SEM of three independent experiments (*n* = 3) is used to express the data. (*) *p* ≤ 0.05 indicates significant differences compared to the control. CRL: control.

Western blot analysis was performed to determine the effect of T-2 on the expression levels of inflammatory and oxidative proteins (Figure 3 and Figure S1). Band detection indicates the presence of β-actin (44 kDa), IL-1β (31 kDa), IL-6 (19 kDa), TNF-α (60 kDa), Nrf2 (26 kDa), and HO-1 (30 kDa) proteins (Figure 3a–c). The β-actin was used as a loading control, and the expression levels of inflammatory and oxidative-stress-related proteins were normalized accordingly. A significant increase in IL-1β expression was observed at the highest T-2 concentration (30 nM), with a 1.56 ± 37-fold change compared to the control (*p* = 0.037) (Figure 3b,d). The IL-6 bands showed a 2.95 ± 0.25-fold increase (*p* = 0.009) (Figure 3b,e), while TNF-α expression increased by 1.62 ± 0.10-fold (*p* = 0.011) (Figure 3f).

Regarding oxidative-stress-related proteins, Nrf2 expression decreased by 0.33 ± 0.12-fold relative to the control (*p* = 0.039) (Figure 3a,g), whereas HO-1 significantly increased up to 3.31 ± 0.23-fold (*p* = 0.001) compared to the control, indicating an adaptive antioxidant response to the oxidative damage induced by T-2 (Figure 3c,h).

## 3. Discussion

In our previous study, T-2 produced an increase in ROS levels in HepG2 cells, accompanied by an upregulation of antioxidant enzymes such as glutathione-S-transferase (GST) and catalase (CAT) [22]. However, the specific molecular pathways involved in ROS generation by T-2 have not yet been specified. Studying the oxidative pathway is important since redox imbalance is the main pathway that causes mitochondria-dependent apoptosis, which is related to disease progression and cell death [23]. This work aims to evaluate the effects of T-2 on the production of proinflammation cytokine and oxidative stress markers in HepG2 cells by qPCR and Western blot analysis. To the best of the authors’ knowledge, no previous studies have simultaneously addressed both aspects in this cell line. Thus, this research contributes to a deeper comprehension of the mechanisms underlying the oxidative damage and inflammation of T-2 in liver cells, to better assess the risk associated with T-2 exposure. Therefore, to demonstrate how the oxidative stress in T-2-induced hepatotoxicity is implicated, the effect of this mycotoxin on Nrf2 and HO-1 protein expression levels was investigated.

It is well known that T-2 produces damage in different cells, such as intestinal, hepatic and neuronal [17,23,24]. The present study examined the marked transcriptional activation of proinflammatory cytokines, such as TNFα, IL-1β, and IL-6. The increase in the gene expression of these cytokines suggests that T-2 induces inflammation in HepG2 cells. This inflammatory response appears to be associated with oxidative stress, as indicated by the decreased expression of Nrf2, a key transcription factor regulating the antioxidant defense system, and the moderate induction of HO-1 at lower concentrations. The coordinated regulation of these genes indicates that both the inflammatory and oxidative pathways are activated in response to T-2 exposure, and that these pathways may act in a synergistic rather than independent manner. These observations align with previous findings in different cellular systems. Pei and coworkers also analyzed the cytokine secretion after 6.43–25.72 µM of T-2 exposure in PC12 cells, a neuronal cell model. They observed an increase in the TNFα, IL-1β, and IL-6 cytokines using a Luminex liquid cytokine chip assay [23]. Similarly, Wang and colleagues reported that T-2 induced the overexpression of proinflammatory genes and upregulated the mRNA expression of TNFα, IL-1β, and IL-6, leading to the activation of the JAK-STAT signaling pathway contributing to immunotoxicity [25]. Janik-Karpinska and coworkers also found an increase in the relative mRNA expression of TNFα and IL-1β after 100–10,000 nM of T-2 exposure in a human foreskin fibroblast line (Hs68 cells) [26]. Similarly, other authors reported that trichothecenes such as T-2 (5 nM) and DON (1 µM) increased the concentrations of the cytokines IL-6 and IL-8 in the intestinal cells HIEC-6 and IPEC-J2, by the ELISA sandwich technique [17,18]. Finally, Toutounchi and collaborators reported that DON (from 4 to 8 µM) and T-2 (from 2 to 8 nM) significantly increased the mRNA level of IL-6, but not IL-1β, in a human placenta choriocarcinoma (BeWo) cell line as shown by qPCR [2].

At the protein level, our Western blot results corroborated the transcriptional findings by qPCR, confirming the upregulation of HO-1 and proinflammatory cytokines. However, a marked suppression of Nrf2 was observed at the highest T-2 concentration. These results are consistent with studies by other authors, such as Zhang and colleagues, who reported an inhibition of the HO-1 and Nrf2 protein upon exposure to T-2 (10.72–171.48 µM) in N2a cells [27]. Interestingly, other studies have reported that low concentrations of T-2 may transiently increase HO-1 expression, possibly as a compensatory mechanism to counteract oxidative damage. However, this protective effect appears to diminish at higher toxin concentrations [23]. Consistent with our findings, those studies also observed a reduction in both Nrf2 and HO-1 levels at elevated T-2 concentrations. The reduced or inhibited levels of Nrf2 aggravate T-2-induced cytotoxicity, because antioxidant mechanisms are not activated [23]. Our findings confirm that T-2 induces oxidative stress in HepG2 cells and the Nrf2/HO-1 pathway is modulated in a concentration-dependent manner. At low concentrations, this pathway appears to be activated as a compensatory response to oxidative damage; however, at higher concentrations, its suppression compromises the antioxidant defense system, thereby contributing to increased inflammation, morphological alterations, and apoptosis.

In addition to the Nrf2/HO-1 pathway studied in this work, there are other common oxidative or inflammatory pathways. The NF-κB is a major signaling pathway, which is activated in response to oxidative stress and plays a critical role in cell proliferation, differentiation, and inflammation [28]. Previous studies demonstrated the implication of NF-κB in the hepatotoxicity induced by T-2 [29]. Likewise, the JAK/STAT pathway is a very common and sensitive signaling pathway in molecular immune toxicity, and is closely related to the proinflammatory cytokine function. Previous studies have reported that the T-2 toxin activated the phosphorylation of the JAK/STAT pathway in RAW264.7 cells [30]. On the other hand, another significant signaling system in cell biology that is crucial for controlling processes including stress, apoptosis, differentiation, and proliferation is the mitogen-activated protein kinase (MAPK) signaling pathway. JNK, p38 protein kinases, and extracellular regulated protein kinases (ERK) are all members of the MAPK family [31]. Although some authors have studied its activation after exposure to T-2 in different cell types [12,32,33,34], its role in liver cells remains unexplored. Therefore, future assays could be conducted in this direction to increase the knowledge of the mechanisms of action of T-2. Additional evidence from recent studies in HepG2 cells supports the role of multiple stress-related signaling pathways in hepatotoxicity. For instance, Zhang and colleagues demonstrated that apoptosis induced by bakuchiol involves the activation of the Bcl-2/Bax/Cyt-c/Caspase-3 axis in HepG2 cells [35]. Similarly, Wu and coworkers reported that hypoxia and pharmacological agents modulate proliferative and invasive behaviors in HepG2 cells through mechanisms involving MAPK and inflammatory pathways [36]. These findings support our results and highlight the relevance of HepG2 cells as a model for studying the interplay between oxidative stress, inflammation, and cell death in response to the T-2 toxin.

To date, no studies have reported morphological changes in HepG2 cells after T-2 exposure. Then, to assess whether the alterations in proinflammatory cytokine expression and the modulation of the Nfr2/HO-1 pathway are related to the morphological changes, this study evaluated potential structural changes after 24 h of treatment with different concentrations of the T-2 toxin, using indirect immunofluorescence. Figure 1a–e visually shows a concentration-dependent morphological change in HepG2 cells, transitioning from a rounded to an elongated shape after T-2 exposure. Upon quantification, Figure 1f reveals that, after treatment with all T-2 concentrations, the majority of cells fell within the 20 to 40 µm length range, in contrast to the control group, where most cells measured between 0 to 20 µm. However, as T-2 concentration increased, a growing proportion of cells exhibited lengths between 80 to 120 µm, a size range not observed at lower toxin concentrations. These findings confirm a clear morphological alteration in response to T-2, likely reflecting underlying cellular damage associated with its toxic effects.

Emerging imaging technologies for high-throughput toxicology screening have been described, such as the neuromorphology-enabled video-activated cell sorter (NEVACS) [17]. It is designed to achieve high-dimensional spatiotemporal characterization. It may be a useful non-invasive platform for detecting toxin-induced changes in cell morphology.

Given that T-2 promotes the expression of proinflammatory cytokines in liver cells, regulating these inflammatory mediators may represent a viable strategy to reduce liver damage. One potential approach involved the use of natural compounds capable of enhancing antioxidant enzyme activity and modulating anti-inflammatory signal pathways, thereby mitigating the toxic effects of T-2 exposure. For instance, Qiao and colleagues evaluated the anti-inflammatory effects of *Rehmannia glutinosa* polysaccharide (RGP), a perennial herb, on LPS-induced acute liver injury in mice. They observed that after RGP treatment, the gene and protein expression levels of proinflammatory factors such as TNF-α, IL-1β, IL-6, and caspase-1 were reduced, whereas the gene expression levels of the anti-inflammatory factor IL-10 were upregulated [37].

Li and coworkers studied an advanced antioxidant and anti-inflammatory therapeutic strategy, which could complement traditional treatments based on natural products. This strategy consists on the creation of self-derived polymorphonuclear neutrophils. In their study, these neutrophils, when injected into mice with sepsis, rapidly migrated to inflamed damaged organs, where they effectively neutralized proinflammatory cytokines and eliminated ROS [38]. This innovative technology shows evident anti-inflammatory and antioxidant effects and holds promising potential for clinical applications.

## 4. Conclusions

In conclusion, the present study demonstrates that T-2 exposure in HepG2 cells leads to an increase in proinflammatory cytokines, especially at high concentrations, such as TNFα (at 7.5, 15, and 30 nM T-2), IL-6 (15 and 30 nM of T-2), and IL-1β (30 nM T-2). At lower T-2 concentrations (7.5 nM), activation of the Nrf2/HO-1 pathway appears to represent a cellular adaptative response to oxidative stress induced by T-2. However, at higher concentrations (15 and 30 nM of T-2), these protective mechanisms are suppressed due to the high cytotoxicity exerted by T-2, which surpasses the cell’s antioxidant defense capacity. The concurrent upregulation of inflammatory markers under these conditions suggests a potential synergistic interaction between oxidative stress and inflammation, both contributing to T-2-induced hepatotoxicity.

This study provides new insights into the inflammatory and oxidative mechanisms triggered by T-2 in liver cells. In this context, the goal of the present study is to serve as a springboard for upcoming research directions that will help to define the mechanism of action (MoA) underlying T-2-induced hepatotoxicity and to support the development of cytoprotective strategies aimed at reducing inflammation and liver damage.

## 5. Materials and Methods

### 5.1. Reagents

Cell culture and analytical-grade reagents, including Dulbecco’s Modified Eagle’s Medium (DMEM), penicillin, streptomycin, trypsin/EDTA, Phosphate-Buffered Saline (PBS), Newborn Calf Serum (NBCS), DiMethyl SulfOxide (DMSO), methylthiazolte-trazolium salt (MTT) dye, and the nuclear stain DAPI, were obtained from Sigma-Aldrich (St. Louis, MO, USA). Methanol (MeOH) was provided by Merck Life Science S.L. (Madrid, Spain), while ultrapure water (resistivity < 18 MΩ·cm) was produced using a Milli-Q purification system (Millipore, Bedford, MA, USA). Western blot reagents and materials were purchased from Bio-Rad Laboratories (Hercules, CA, USA).

The primary monoclonal rabbit β-actin antibody (4970), anti-rabbit horseradish peroxidase (HRP)-linked (7074), and anti-mouse horseradish peroxidase (HRP)-linked (7076) were obtained from Cell Signaling Technology (Danvers, MA, USA). Polyclonal rabbit HO-1 (10701-1-AP) and monoclonal mouse TNF-α (60291-1-Ig) were purchased by Proteintech (Chicago, IL, USA). Monoclonal mouse Nrf2 (A-10, sc-365949) was purchased from Santa Cruz Biotechnology (Santa Cruz, CA, USA). Monoclonal mouse IL-6 (MAB206) was purchased by R&D Systems (Minneapolis, MN, USA). Polyclonal rabbit IL-1β (P420B) was acquired from Invitrogen (Carlsbad, CA, USA). Polyclonal chicken β-actin antibody (251-006) was obtained by Synaptic Systems (Göttingen, Germany). The secondary antibody Alexa Fluor 647^®^ Donkey Anti-Chicken (703-605-155) was purchased by Jackson ImmunoResearch (West Grove, PA, USA).

The standard of T-2 (MW:466.52 g/mol) was purchased from Sigma-Aldrich (St. Louis, MO, USA). The stock solution of the mycotoxin was prepared in MeOH at the appropriate working concentration and subsequently maintained in a dark environment at −20 °C.

### 5.2. Cell Culture and Treatment

The human hepatocarcinoma (HepG2) cells (ATCC: HB-8065) were maintained in DMEM supplemented with 10% newborn calf serum (NBCS), 100 U/mL penicillin, and 100 mg/mL streptomycin. Cells were incubated at 37 °C in a humidified atmosphere containing 5% CO_2_ and 95% air, at physiological pH (7.4) and were passaged twice weekly at a 1:3 ratio following enzymatic detachment with trypsin.

In previous studies, the IC_50_ value of T-2 in HepG2 cells was obtained by MTT assay, which was 68.6 ± 4.8 nM [21]. In this work, the concentrations IC_50_/2 (30 nM), IC_50_/4 (15 nM), and IC_50_/8 (7.5 nM) were selected for the assays. These relatively high concentrations were chosen to investigate the acute toxicity of T-2 and to better understand its MoA. Additionally, this study built upon a series of previous investigations in which the same concentrations were used, ensuring the continuity and comparability of results. The final concentrations of mycotoxin were achieved by the addition of T-2 to the culture medium, with a final MeOH concentration of ≤1% (*v*/*v*). The results were compared between cells cultured in medium alone and in medium containing ≤1% methanol, showing no statistically significant differences between both conditions. It is imperative to note that each experiment incorporated appropriate controls, comprising an equivalent amount of solvents.

### 5.3. Immunofluorescence Detection

Immunofluorescence was used to analyze the morphological changes in HepG2 cells after T-2 treatment. β-actin (1:500 dilution) was used as a structural marker to visualize the cytoskeleton and assess morphological integrity. This staining allowed the observation of changes in cell shape and organization, potentially associated with T-2-induced cytoskeletal alterations and cellular stress. For the immunofluorescence assay, 2 × 10^4^ HepG2 cells were seeded in a 12-well chamber (Ibidi, GmbH, Gräfelfing, Germany #81201). Following a 24 h incubation period, the cells were exposed to different concentrations of T-2 (IC_50_/2, IC_50_/4 and IC_50_/8) and a control (≤1% MeOH) for a further 24 h. Then, the cells were washed with PBS and subsequently fixed with 4% formaldehyde for 1 h at room temperature. Following fixation, the solution was removed, and the cells were washed three times with PBS under gentle agitation at room temperature. The cells were subjected to permeabilization and blocking in a working solution (1 mL of 0.3% Triton X100, 20% horse serum, and 2% BSA in PBS) and incubated overnight. After 24 h, the cells were washed with PBS and then incubated overnight with the primary antibody β-actin (1:500), diluted in the working solution with 0.2% Triton X-100, at 4 °C. The next day, after some PBS washes, the cells were incubated with a secondary antibody (Alexa Fluor^®^ 647) and DAPI (1:500), which had been diluted in the working solution with 0.2% Triton X-100, for 3 h at room temperature. Cells were washed with PBS and images were captured using an Olympus Fluoview FV1000 confocal laser scanning microscope (Olympus, Tokyo, Japan), equipped with a UPLSAPO 20× objective lens (numerical aperture: 0.75). Alexa Fluor^®^ 647 was excited using a 633 nm laser, and fluorescence emission was collected in the 660–700 nm range. The excitation of DAPI was performed at 405 nm, and the emission was collected at 450–470 nm. All images were captured at a resolution of 1024 × 1024 pixels. Exposure time and gain settings were kept constant across all samples to ensure the valid comparison of fluorescence intensity and morphology between conditions.

### 5.4. RNA Extraction and Quantification

To investigate whether the observed cytotoxicity was associated with inflammatory or oxidative stress pathways, the levels of the expression of related genes in HepG2 cells were evaluated. These cells were seeded at a density of 47.2 × 10^4^ cells/well in a 6-well plate.

Upon reaching approximately 80% confluence, the cells were exposed to fresh culture medium supplemented with 7.5, 15, or 30 nM of T-2 for a 24 h. Total RNA was then extracted using the ReliaPrep™ RNA Cell Miniprep System (Promega, Madison, WI, USA), followed by RNase-free DNase (Promega, Madison, WI, USA) to remove any genomic DNA contamination. The extracted RNA of each condition was initially subjected to a quantity and quality assessment using a Nanodrop 2000 (Thermo Fisher Scientific, Madrid, Spain). This analysis provides concentrations ranging from 186.8 to 401.1 ng/μL, with appropriate 260/280 and 260/230 ratios, to indicate protein and phenol/salt/EDTA/non-ionic detergent contamination, respectively, both ≥2. The nanodrop has been widely used for evaluating the quality of extracted RNA in recent literature [39,40,41,42,43]. Isolated RNA was stored at −20 °C. Then, the samples were diluted to a concentration of 100 ng/μL with pure Milli-Q water, to facilitate reverse transcription to complementary DNA (cDNA). Three biological replicates were carried out. After that, these samples were mixed with the MIX of TaqMan™ MicroRNA Reverse Transcription Kit (Thermo Fisher Scientific, Madrid, Spain) for cDNA synthesis, with an amount of 5000 ng of RNA per condition.

### 5.5. Reverse Transcription and qPCR Reaction

Complementary DNA (cDNA) was synthesized from 5 μL of total RNA using the TaqMan™ MicroRNA Reverse Transcription Kit (Thermo Fisher Scientific, Madrid, Spain), according to the manufacturer’s instructions. The relative IL-1β, IL-6, Nrf2, TNF-α, and HO-1 mRNA expression was measured in HepG2 cells. Each gene was amplified by qPCR using specific primers, and product specificity was validated by the melting curve method. A single sharp peak was observed for each primer pair. Primer pairs for each target gene were designed using the Primer-BLAST tool (NCBI website, accessed January 2025), following established guidelines and considering the full coding sequence of each candidate gene as reference. Design criteria included primers of approximately 20 nucleotides, a GC content between 45% and 60%, melting temperatures (Tm value) ranging from 58 °C to 60 °C, and expected amplicon sizes between 50 and 150 base pairs. The efficiency of each primer set was evaluated through a standard curve, prepared from a two-fold serial dilution of cDNA. All qPCR reactions were performed in triplicate to ensure reproducibility. The primers used for gene amplification are detailed in Table 1. Real-time amplification reactions were conducted within 96-well plates using a SYBR Green detection reactive, with subsequent analysis facilitated by the StepOne Plus Real-time PCR instrument (Applied Biosystems, Foster City, CA, USA). Each reaction was carried out in a final volume of 10 μL, which included 3 μL of cDNA template diluted 1:2 (1000 ng cDNA/condition), 2 μL of amplification primer mix (forward/reverse of each gene; 2.5 μM), and 5 μL of SYBR Green. In addition, non-template controls (NTC) were included for each primer pair, the template being replaced by DNAse- and RNAse-free water. Thermal cycling conditions were set using default settings, starting with an initial denaturation at 95 °C for 5 min to activate Taq DNA polymerase. This was followed by 40 amplification cycles consisting of denaturation at 95 °C for 15 s, annealing at 58–60 °C (primer-dependent) for 15 s, and elongation at 72 °C for 45 s. A melting curve analysis was generated by gradually increasing the temperature from 60 °C to 90 °C. The presentation of melting curves in Figure S2 confirms the absence of non-specific amplifications, providing an additional level of quality control. Therefore, even if only one reference gene (rS18) was analyzed, these melting curves allow for the validation of the reliability of the observed amplification, ensuring that the results are based on target-specific amplification. Threshold cycles (Ct) and primer parameter analysis were automatically determined using the StepOne Plus software version 2.4 (Applied Biosystems). The average of the target gene values was normalized to the reference gene rS18 value and expressed as fold changes (2^−ΔΔCt^) compared with the solvent control.

### 5.6. Western Blot Analysis

For the Western blot assay, proteins were obtained from HepG2 cells after T-2 exposure. Cells were lysed and proteins were extracted in 200 μL of RIPA buffer supplemented with the protease inhibitor cocktail and phosphatase inhibitor cocktail provided in the kit (#sc-24948, Santa Cruz Biotechnology, Santa Cruz, CA, USA). Samples were centrifuged at 12,000× *g* for 15 min at 4 °C to isolate cellular proteins from the supernatant fraction. Protein concentration was determined using the Bradford assay (BioRad Laboratories, Madrid, Spain) with Cydex modification [44]. A standard curve was generated using serial dilutions of bovine serum albumin (BSA), and blank controls were included to ensure assay accuracy. Equal amounts of proteins (25 µg per sample) were separated by 7.5% sodium dodecyl sulphate-polyacrylamide gel electrophoresis (SDS-PAGE) and transferred to a 0.2 µm polyvinylidene difluoride (PVDF) membrane (BioRad Laboratories, Madrid, Spain). Membranes were incubated in 5% (*w*/*v*) non-fat dry milk prepared in TBS-T buffer for 1 h at room temperature to block non-specific binding, followed by incubation with rabbit HO-1 (1:1000), mouse TNF-α (1:500), mouse Nrf2 (1:200), mouse IL-6 (1:500), rabbit IL-1β (1:500), or rabbit anti-β-actin (1:1000) in blocking buffer at 4 °C overnight. Then, the membranes were washed three times with TBS-Tween buffer, blocked for 1 h and incubated with anti-mouse or anti-rabbit HRP-conjugated secondary antibodies (1:5000), depending on the primary antibody, for 2 h at room temperature. Protein bands were detected using chemiluminescence (ECL) reagents (Thermo Fisher Scientific, MA, USA) and quantified with ImageJ software (version 1.54g; NIH, Bethesda, MD, USA). For each sample, the intensity of the band corresponding to the protein of interest was measured and corrected for background. This value was then normalized to the β-actin band intensity from the same lane. To facilitate comparison, the normalized value of the control group was set as 1, and treated samples were expressed as fold changes relative to this internal reference. This approach ensures consistency with the qPCR data presentation.

### 5.7. Statistical Analysis

Statistical analysis of the data was carried out with the Statgraphics statistical package version 16.01.03 (IBM Corp., Armonk, NY, USA). The mean ± SEM of three independent experiments (*n* = 3) was used to express the data: *n* = 3 biological replicates and *n* ≥ 3 technical replicates. Statistical analysis of the results was performed using Student’s *t*-test for paired samples. Prior to the ANOVA analysis, the normality of the data was assessed using the Shapiro–Wilk test and subsequently analyzed using the appropriate statistical methods. Group differences were evaluated using one-way ANOVA, followed by Tukey’s HSD post-hoc test for multiple comparisons. A *p*-value of ≤ 0.05 was considered statistically significant. The statistical power of the analysis of this work was considered using an alpha threshold of 0.05. Therefore, all *p*-values below 0.05 were considered statistically significant, sufficient to reject the null hypothesis and accept the alternative hypothesis.

## Figures and Tables

**Table 1:** Gene-specific primers for qPCR assays.

RefSeq Accession (NM_)	Gene Symbol	Gene Name	Optimum Tª (°C)	Forward Primer/Revers Primer
NM_000576.3	IL-1β	Interleukin-1 β	60	5′-GGAGAATGACCTGAGCACCT-3′ 3′-GGAGGTGGAGAGCTTTCAGT-5′
NM_00600.5	IL-6	Interleukin-6	60	5′-AGTCCTGATCCAGTTCCTGC-3′ 3′-CTACATTTGCCGAAGAGCCC-5′
NM_00594.4	TNF-α	Tumor Necrosis Factor-α	58	5′-CCGACTATCTCGACTTTGCC-3′ 3′-ACAGGGCAATGATCCCAAAG-5′
NM_001145412.3	Nrf2	Nuclear factor erythroid type 2	62	5′-TCCAGTCAGAAACCAGTGGAT-3′ 3′-GAATGTCTGCGCCAAAAGCTG-5′
NM_002133.3	HO-1	Heme oxygenase-1	60	5′-AAGACTGCGTTCCTGCTCAAC-3′ 3′-AAAGCCCTACAGCAACTGTCG-5′
NM_022551.3	rS18	Ribosomal S18	58	5′-CGGCTACCACATCCAAGGAA-3′ 3′-GCTGGAATTACCGCGGCT-5′

## Data Availability

The original contributions presented in this study are included in this article and Supplementary Materials. Further inquiries can be directed to the corresponding author.

## Supplementary Materials

The following supporting information can be downloaded at: https://www.mdpi.com/article/10.3390/toxins17080397/s1, Figure S1: Original western blot images showing the expression levels of TNF-α, Nrf2, HO-1 and IL-1β, IL-6 proteins in lysates of HepG2 cells exposed to T-2 (7.5, 15 and 30 nM) for 24 h, with the corresponding β-actin reference protein. Figure S2: Melting curve analysis of genes rS18, TNF-α, IL-1β, IL-6, Nrf2 and HO-1 in HepG2 cells exposed to T-2 (7.5, 15 and 30 nM) for 24 h obtained by StepOne Plus software version 2.4.

## Abbreviations

The following abbreviations are used in this manuscript:

CAT	catalase
cDNA	complementary DNA
DAPI	4′,6-diamidine-2′-phenylindole dihydrochloride
DMEM	Dulbecco’s Modified Eagle’s Medium
DMSO	diMethyl SulfOxide
ELISA	Enzyme-Linked Immunosorbent Assay
GPx	Glutathione peroxidase
GST	Glutathione-S-transferase
HO-1	Heme oxygenase-1
IL-1β	Interleukin-1 β
IL-6	Interleukin-6
IL-8	Interleukin-8
kDa	kilodaltons
mRNA	Messenger RNA
MTT	Methylthiazolte-trazolium salt
NBCS	Newborn Calf Serum
NQO1	NAD(P)H quinone dehydrogenase 1
Nrf2	Nuclear factor erythroid type 2
PBS	Phosphate-Buffered Saline
PVDF	Polyvinylidene difluoride
qPCR	quantitative polymerase chain reaction
ROS	Reactive oxygen species
SOD	Superoxido dismutase
T-2	T-2 toxin
Tm	melting temperature
TNF-α	Tumor Necrosis Factor-α
