# Supplementary material for: T-2 Toxin-Induced Hepatotoxicity in HepG2 Cells Involves the Inflammatory and Nrf2/HO-1 Pathways
Source: Toxins (Basel). 2025 Aug 8;17(8):397. doi: 10.3390/toxins17080397 (PMC12390224; doi:10.3390/toxins17080397)
Supplement: Supplementary file 1 [file toxins-17-00397-s001.zip › toxins-3750070-supplementary.pdf]

SUPPLEMENTARY MATERIAL

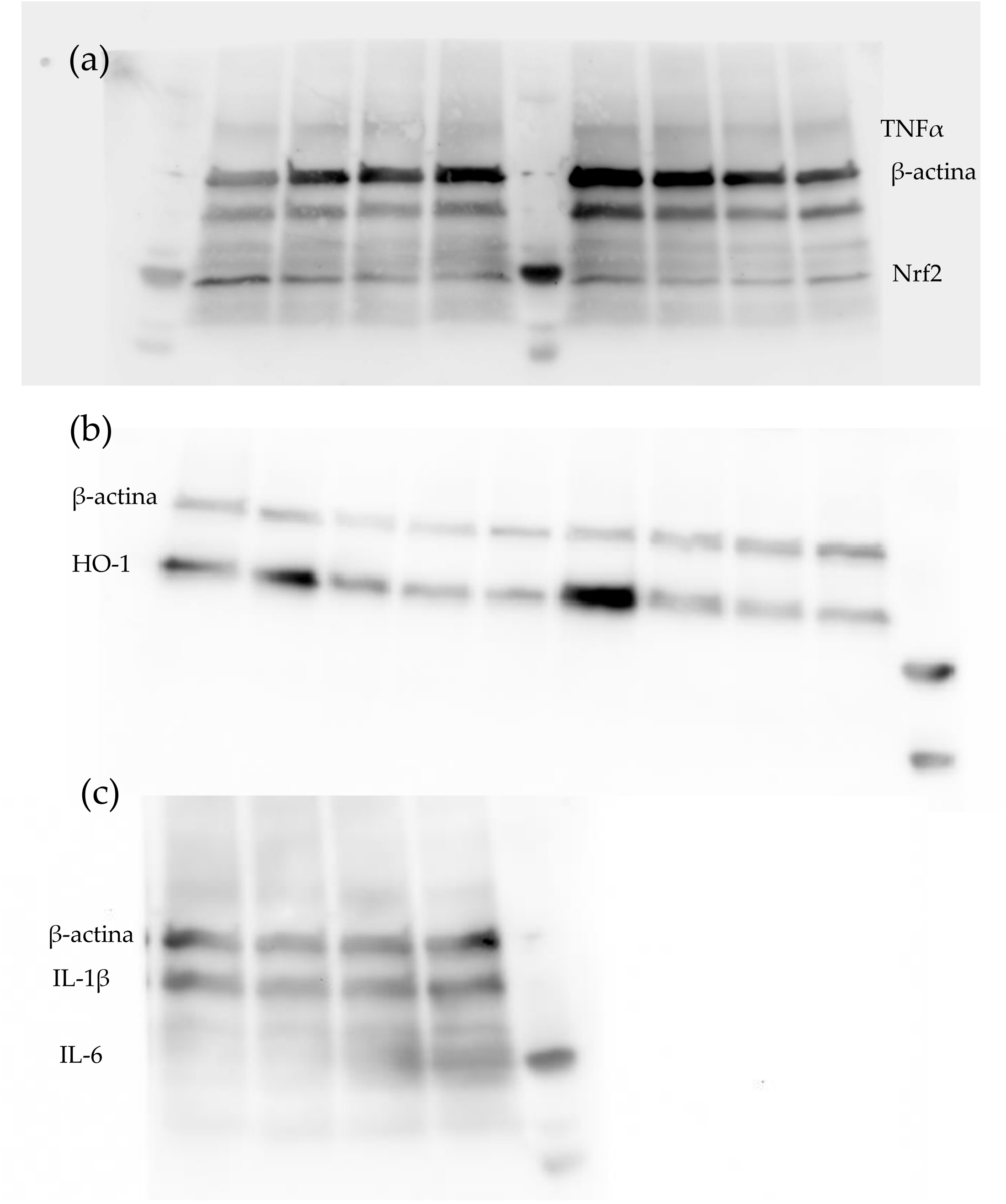

Figure S1. Original western blot images showing the expression levels of TNF-α, Nrf2 (a), HO-1 (b) and IL-1β, IL-6 (c) proteins in lysates of HepG2 cells exposed to T-2 (7.5, 15 and 30 nM) for 24 h, with the corresponding β-actin reference protein. In a) the last four lanes correspond to the analyzed condition. In b) the first four lanes correspond to the analyzed condition.

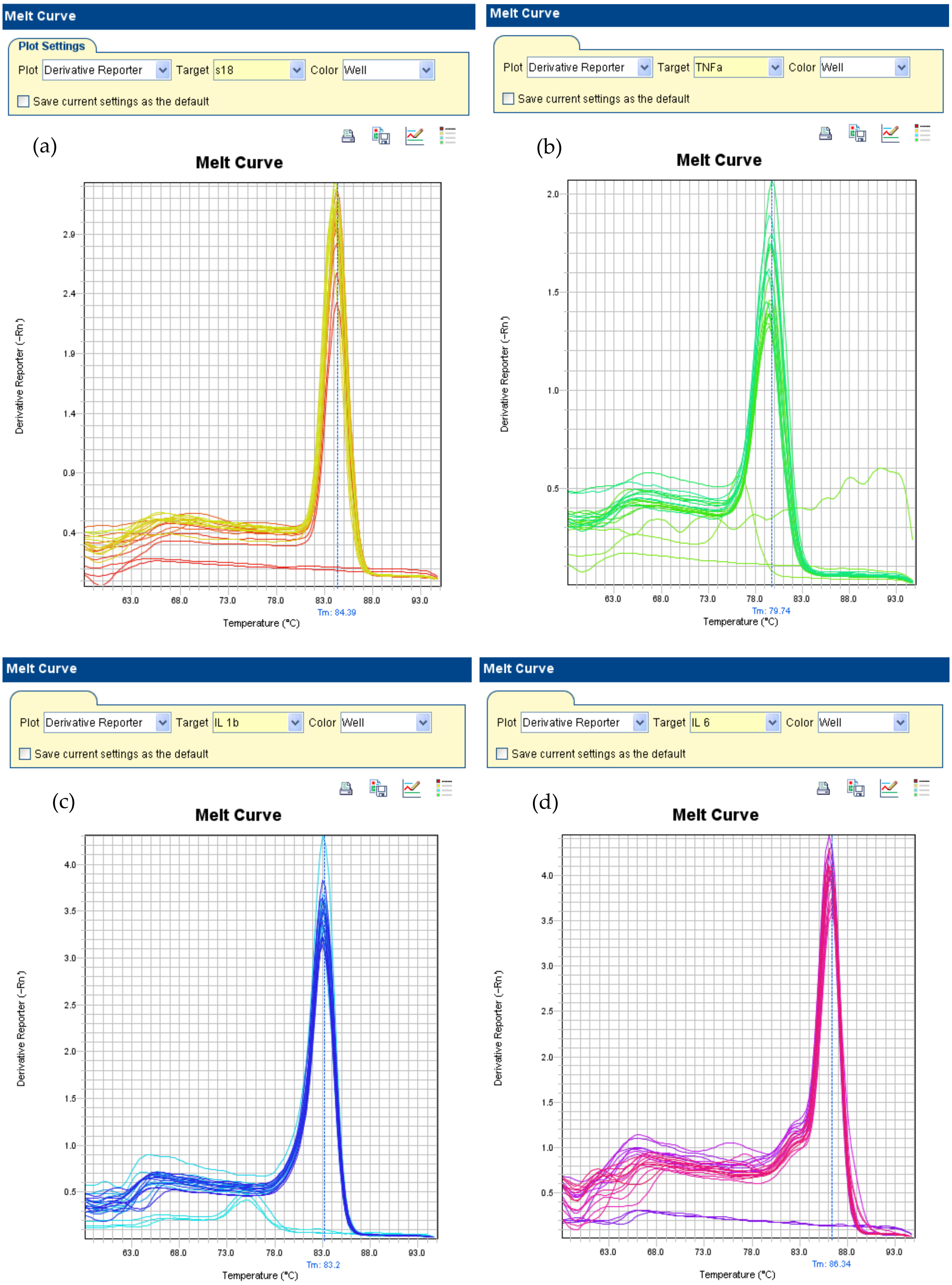
Melt Curve
Plot Settings
Plot Derivative Reporter
Target s18
Color Well
Save current settings as the default
(a)
Melt Curve
Derivative Reporter (-Rn')
Temperature (°C)
Tm: 84.39
Melt Curve
Plot Derivative Reporter
Target TNFa
Color Well
Save current settings as the default
(b)
Melt Curve
Derivative Reporter (-Rn')
Temperature (°C)
Tm: 79.74
Melt Curve
Plot Derivative Reporter
Target IL 1b
Color Well
Save current settings as the default
(c)
Melt Curve
Derivative Reporter (-Rn')
Temperature (°C)
Tm: 83.2
Melt Curve
Plot Derivative Reporter
Target IL 6
Color Well
Save current settings as the default
(d)
Melt Curve
Derivative Reporter (-Rn')
Temperature (°C)
Tm: 86.34

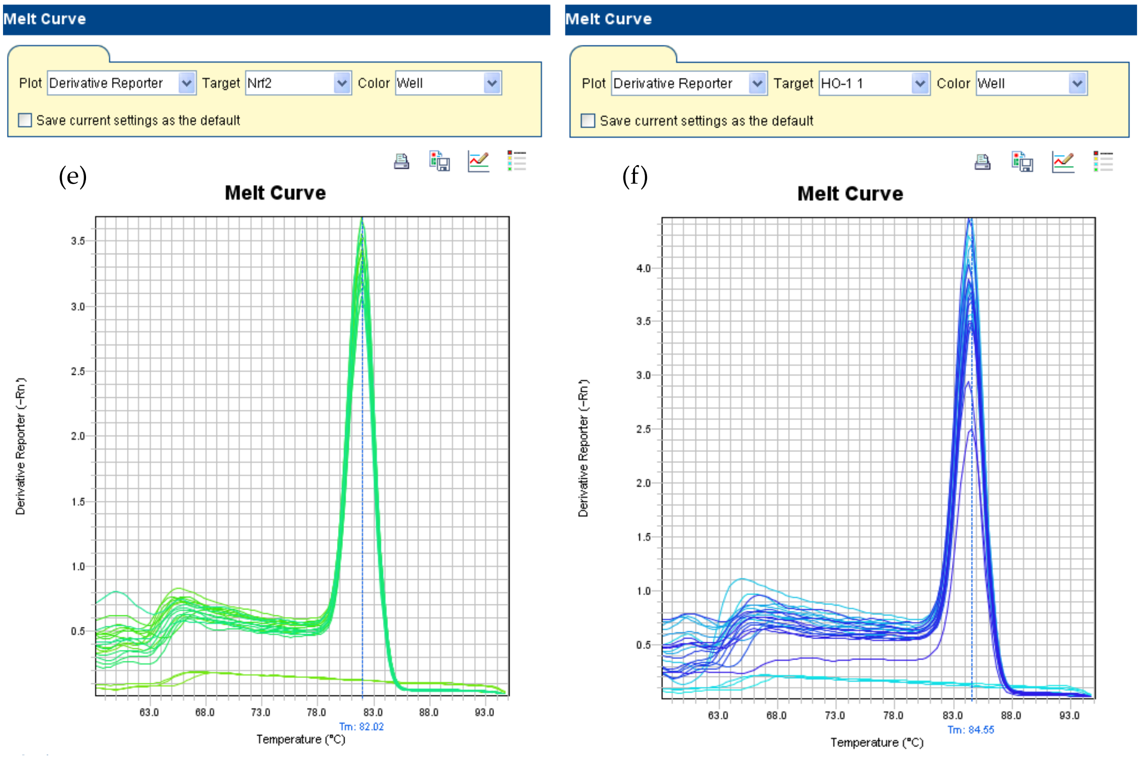

Figure S2. Melting curve analysis of genes rS18 (a), TNF-α (b), IL-1β (c), IL-6 (d), Nrf2 (e) and HO-1 (f) in HepG2 cells exposed to T-2 (7.5, 15 and 30 nM) for 24 h obtained by StepOne Plus software version 2.4 (Applied Biosystems).
